# Mitochondrial fatty acid β-oxidation is important for normal osteoclast formation in growing female mice

**DOI:** 10.3389/fphys.2022.997358

**Published:** 2022-09-14

**Authors:** Priyanka Kushwaha, Nathalie S. Alekos, Soohyun P. Kim, Zhu Li, Michael J. Wolfgang, Ryan C. Riddle

**Affiliations:** ^1^ Department of Orthopaedic Surgery, Johns Hopkins University School of Medicine, Baltimore, MD, United States; ^2^ Department of Orthopaedics, University of Maryland School of Medicine, Baltimore, MD, United States; ^3^ Department of Biological Chemistry, Johns Hopkins University School of Medicine, Baltimore, MD, United States; ^4^ Baltimore Veterans Administration Medical Center, Baltimore, MD, United States

**Keywords:** bone, osteoclast, lipid metabolism, CPT2, glucose

## Abstract

Skeletal remodeling is an energy demanding process that is linked to nutrient availability and the levels of metabolic hormones. While recent studies have examined the metabolic requirements of bone formation by osteoblasts, much less is known about the energetic requirements of bone resorption by osteoclasts. The abundance of mitochondria in mature osteoclasts suggests that the production of an acidified micro-environment conducive to the ionization of hydroxyapatite, secretion of matrix-degrading enzymes, and motility during resorption requires significant energetic capacity. To investigate the contribution of mitochondrial long chain fatty acid β-oxidation to osteoclast development, we disrupted the expression of carnitine palmitoyltransferase-2 (Cpt2) in myeloid-lineage cells. Fatty acid oxidation increases dramatically in bone marrow cultures stimulated with RANKL and M-CSF and microCT analysis revealed that the genetic inhibition of long chain fatty acid oxidation in osteoclasts significantly increases trabecular bone volume in female mice secondary to reduced osteoclast numbers. In line with these data, osteoclast precursors isolated from Cpt2 mutants exhibit reduced capacity to form large-multinucleated osteoclasts, which was not rescued by exogenous glucose or pyruvate, and signs of an energetic stress response. Together, our data demonstrate that mitochondrial long chain fatty acid oxidation by the osteoclast is required for normal bone resorption as its inhibition produces an intrinsic defect in osteoclast formation.

## Introduction

Bone resorption by the osteoclast is essential for the maintenance of skeletal integrity and the liberation of mineral ions stored in bone tissue. In response to macrophage colony-stimulating factor (M-CSF) and receptor activator of nuclear factor-κΒ ligand (RANKL) (Yoshida et al., 1990; Lacey et al., 1998) produced by osteoblasts, the monocyte/macrophage precursors that give rise to osteoclasts are recruited to bone surfaces where they fuse to form the multinucleated polykaryon. Contact with bone matrix *via* α_v_β_3_ integrins (McHugh et al., 2000) and their subsequent polarization and formation of a ruffled membrane allow the osteoclast to form a resorption cavity necessary for their unique function (Blair et al., 1989).

Acidification of the resorption cavity, the secretion of proteolytic enzymes, and migration of osteoclasts within cutting cones are expected to require significant energetic capacity (Arnett and Orriss, 2018), but the intermediary metabolism of these cells has not been well studied. *In vitro* studies wherein avian or rodent osteoclasts were cultured on tissue culture plastic or bone fragments suggest that osteoclasts are glycolytic (Williams et al., 1997; Larsen et al., 2002; Larsen et al., 2005; Kim et al., 2007). RANKL treatment increases glucose uptake and lactate production (Kim et al., 2007) and knockdown of lactate dehydrogenase A or B (LDHA, LDHB) impairs precursor fusion (Ahn et al., 2016). Moreover, biochemical and cellular localization studies suggest that the v-ATPase responsible for proton secretion interacts with glycolytic enzymes including aldolase and phosphofructokinase (Lu et al., 2001; Su et al., 2003), which raises the possibility that localized ATP production *via* glucose catabolism fuels matrix demineralization.

Additional studies highlight a requirement for mitochondrial respiration to drive osteoclast formation and activity. Osteoclasts have a well-defined mitochondrial network (Chuan, 1931) and treating osteoclast precursors with rotenone to inhibit Complex I of the electron transport chain reduces osteoclast differentiation and resorption both *in vitro* and in a mouse model of induced osteolysis (Kwak et al., 2010). RANKL stimulation has been reported to increase the rate of oxygen consumption even as it promotes glycolysis (Kim et al., 2007; Ahn et al., 2016) and to drive mitochondrial biogenesis (Ishii et al., 2009; Zhang et al., 2018). These effects on oxidative metabolism are mediated by peroxisome proliferator-activated receptor-gamma coactivator 1β and nuclear factor-κΒ signaling (Ishii et al., 2009; Zeng et al., 2015; Zhang et al., 2018) as well as Myc and estrogen receptor–related receptor-α (Bae et al., 2017).

Since active human osteoclasts exhibit a high level of β-hydroxyacyl dehydrogenase activity *in situ* (Dodds et al., 1994) and palmitic acid, the most common saturated fatty acid in animals, increases osteoclast differentiation (Drosatos-Tampakaki et al., 2014; Qu et al., 2018), we explored the requirement for mitochondrial long chain fatty acid β-oxidation in osteoclast development. Long chain fatty acids are taken up by cells by cell surface transporters like members of the SLC27a family and CD36, some of which are expressed by osteoclasts (Dawodu et al., 2018; Koduru et al., 2018). Once inside the cell, fatty acids are conjugated to Coenzyme A and then shuttled into the mitochondrial matrix for oxidation by carnitine acyl-transferase (CPT) enzymes and carnitine-acylcarnitine translocase. CPT1, which is encoded by three different genes (CPT1A, CPT1B, and CPT1C), associates with the outer mitochondrial membrane and catalyzes the conversion of fatty acyl-CoAs to fatty acyl-carnitines that are recognized by the translocase. CPT2, encoded by a single gene, is found on the inner mitochondrial membrane and reverses the actions of CPT1 so that fatty acyl-CoAs can be catabolized (Houten and Wanders, 2010).

Here, we examined skeletal homeostasis and osteoclast formation in a genetic mouse model of myeloid-specific ablation of Cpt2 (Gonzalez-Hurtado et al., 2017). We report that long chain fatty acid β-oxidation increases during osteoclast differentiation *in vitro* and that inhibiting this metabolic pathway greatly diminishes osteoclast formation. *In vivo*, Cpt2 ablation results in increased trabecular bone volume in the femur of female mice by reducing osteoclast numbers. These data suggest that fatty acids represent a key energy source for bone remodeling.

## Materials and methods

Mouse models—The Institutional Animal Care and Use Committee of the Johns Hopkins University approved all procedures using mice. Mice with myeloid-specific ablation of Cpt2 (Gonzalez-Hurtado et al., 2017) were generated by crossing Cpt2^loxP/loxP^ mice (Lee et al., 2015) with lysozyme2-Cre transgenic mice (Clausen et al., 1999). Mice were maintained on a C57BL/6 background; housed on ventilated racks with a 14:10-h light-dark cycle, and fed a standard chow diet (Extruded Global Rodent Diet, Harlan Laboratories).

Skeletal Phenotyping—High-resolution images of the mouse femur were obtained using a desktop microCT scanner (SkyScan 1,275, Bruker) in accordance with the recommendations of the American Society for Bone and Mineral Research (Bouxsein et al., 2010). Bones were scanned at 65 keV and 153 μA using a 0.5 mm aluminum filter with an isotropic voxel size of 10 μm. Trabecular bone parameters were assessed in a region of interest 500 μm proximal to the growth plate and extending for 2 mm (200 CT slices). Femoral cortical bone structure was assessed in a 500-μm region of interest centered on the mid-diaphysis. Bone formation was assessed at 6 weeks of age by intraperitoneal injection of calcein (10 mg/kg) at day 7 and alizarin (30 mg/kg) at day 3 before sacrifice. Dissected femurs were fixed in 4% paraformaldehyde, embedded in methyl methacrylate and then sectioned for histomorphometric analysis. To examine osteoclast numbers, femurs were fixed in a 4% paraformaldehyde solution, followed by decalcification in 14% EDTA for 14 days. From the decalcified samples, 5 uM section were cut and stained for TRAP according to standard techniques. Serum P1NP and CTx were measured by ELISA (Immunodiagnotic systems).

Osteoclast cell culture—Bone marrow cells were isolated from murine long bones of C57BL/6 mice or control and ΔCpt2 and cultured in α-MEM supplemented with 10% fetal bovine serum, penicillin/streptomycin and 50 ng/ml M-CSF. After 24 h of culture, non-adherent cells were collected, centrifuge at 1,500 rpm for 5 min and replated with M-CSF (50 ng/ml). After an additional 24 h of culture, RANKL (50 ng/ml) and M-CSF (50 ng/ml) were added to induce differentiation (Day 0) over the course of 5 days. For rescue experiments, supplemental glucose (0.5 mg/ml), pyruvate (1 mM), or octanoate (0.2 mM) was added directly to the culture medium.

Osteoclast differentiation assays—To quantify osteoclast formation *in vitro*, cultures were fixed and stained for TRAP, using the TRAP-Leukocyte kit (Sigma, St Louis, MO, United States), and then analyzed with ImageJ software. Actin ring formation was assessed by permeabilizing paraformaldehyde fixed cultures with 0.1% Triton X-100 and then staining with phalloidin-FITC (50 μg/ml, Sigma-Aldrich) for 40 min at room temperature. Actin rings were visualized by fluorescent microscopy and analyzed using ImageJ software.

Gene expression analyses—RNA was extracted from osteoclast cultures using TRIzol (Life Technologies). Reverse transcriptase reactions were carried out using 1 μg of RNA and the iScript cDNA Synthesis system (Bio-Rad). qPCR was carried out using primer sequences (Table 1) obtained from PrimerBank (http://pga.mgh.harvard.edu/primerbank/index.html) and iQ Sybr Green Supermix (Bio-Rad). For western blot analysis, cell lysates were resolved by 10% SDS-PAGE, transferred to PVDF membranes and then probed with antibodies for p-AMPKα (Cat no. 2535T); AMPKα, (Cat no. 5831); p-AKT (Cat no. 4060S); AKT (Cat no. 9272S); Phospho-4E-BP1 (Cat no.−2855S); 4E-BP1 (Cat no. 9452); β-actin, (Cat no. 3700) from Cell Signaling Technologies. Mitochondrial proteins were detected *via* the Total OXPHOS rodent antibody cocktail (Abcam, Cat no. ab110413).

**TABLE 1:** qPCR Primers.

Gene	Sequence	
18S	CTT​AGA​GGG​ACA​AGT​GGC​G	ACG​CTG​AGC​CAG​TCA​GTG​TA
Osteoclast differentiation		
Acp5a	CAC​TCC​CAC​CCT​GAG​ATT​TGT	CCC​CAG​AGA​CAT​GAT​GAA​GTC​A
Atp6v0d2	CTG​GTT​CGA​GGA​TGC​AAA​GC	GTT​GCC​ATA​GTC​CGT​GGT​CTG
Ctsk	CTC​GGC​GTT​TAA​TTT​GGG​AGA	TCG​AGA​GGG​AGG​TAT​TCT​GAG​T
Dcstamp	TTT​CCT​ATG​CTG​TTC​CAA​GCG	GCC​GCA​ATC​AAA​GCG​TTC​C
Mmp9	GGA​CCC​GAA​GCG​GAC​ATT​G	CGT​CGT​CGA​AAT​GGG​CAT​CT
Oscar	ACA​CAC​ACA​CCT​GGC​ACC​TA	AGT​CCA​AGG​AGC​CAG​AAC​CT
Nfatc1	GCC​TTT​TGC​GAG​CAG​TAT​CTG	GCT​GCA​CCT​CGA​TCC​GAA​G
Nfkb1	ATG​GCA​GAC​GAT​GAT​CCC​TAC	CGG​AAT​CGA​AAT​CCC​CTC​TGT​T
Tnfrf11a	GCT​CAA​CAA​GGA​TAC​GGT​GTG	AGA​CTG​GGC​AGG​TAA​GCC​T
Traf6	TAC​GAT​GTG​GAG​TTT​GAC​CCA	CAC​TGC​TTC​CCG​TAA​AGC​CAT
Metabolism		
Acaa1a	ACG​CAT​CGC​CCA​ATT​TCT​GA	CCA​GAC​AGG​GAC​ATG​GAC​TC
Acadl	TTT​CCT​CGG​AGC​ATG​ACA​TTT​T	GCC​AGC​TTT​TTC​CCA​GAC​CT
Acadvl	ACT​ACT​GTG​CTT​CAG​GGA​CAA	GCA​AAG​GAC​TTC​GAT​TCT​GCC
Acads	GAC​TGG​CGA​CGG​TTA​CAC​A	GGC​AAA​GTC​ACG​GCA​TGT​C
Acsl1	CTG​ATT​GAC​ATT​CGG​CAG​TAC​G	CCC​CAT​GAG​GGT​GTT​GGT​TG
Cpt1a	TGG​CAT​CAT​CAC​TGG​TGT​GTT	GTC​TAG​GGT​CCG​ATT​GAT​CTT​TG
Cpt2	CCT​GCT​CGC​TCA​GGA​TAA​ACA	GTG​TCT​TCA​GAA​ACC​GCA​CTG
Ldha	CAA​AGA​CTA​CTG​TGT​AAC​TGC​GA	TGG​ACT​GTA​CTT​GAC​AAT​GTT​GG
Ldhb	TGC​GTC​CGT​TGC​AGA​TGA​T	TTT​CGG​AGT​CTG​GAG​GAA​CAA
Hadha	TGC​ATT​TGC​CGC​AGC​TTT​AC	GTT​GGC​CCA​GAT​TTC​GTT​CA
Slc2a1	GCA​GTT​CGG​CTA​TAA​CAC​TGG	GCG​GTG​GTT​CCA​TGT​TTG​ATT​G

*In vitro* metabolic studies—For oxidation experiments, bone marrow cells were seeded in T-25 flasks and treated with M-CSF and RANKL as above. On the day of the experiment, flasks were fitted with rubber stoppers equipped with a well containing Whatman filter paper as previously described (Kim et al., 2017). For fatty acid oxidation cell cultures were incubated at 37°C in media containing 0.5 mM L-carnitine, 0.2% BSA, and 0.06 μCi [1–^14^C]-Oleic Acid (CH_3_(CH_2_)_7_CH = CH(CH_2_)_7_ [^14^C]OOH, Moravek) for 3 h. Expired ^14^CO_2_ was captured and counted by the addition of 1 N perchloric acid to the cell culture medium and 1 M NaOH to the center well containing Whatman filter paper. Oxidation of [1–^14^C]-glucose (HOCH_2_(CHOH)_4_ [14C]HO, Moravek) and [^14^C(U)]-glutamine (Moravek) to ^14^CO_2_ were assessed using the same technique with identical activity levels added to the reaction mix. Cellular ATP content was measured using the ATPlite kit (PerkinElmer) according to the manufacturer’s instructions. Lactate was measured using a Lactate Assay Kit (sigma- MAK064). Glucose consumption by osteoclasts in a 24 h period was measured by subtracting the glucose concentration of culture media from that of blank media using the Glucose (HK) Assay Kit (Sigma-GAHK20). For all metabolic experiments results were normalized to protein concentration determined by the BCA methods (Pierce).

Statistics- Statistical analyses were determined by student t-test or 1-way Anova followed by Tukey’s multiple comparison test as appropriate using Prism software (GraphPad). Results were presented as means ± SEM. *p* value less than 0.05 was considered significant (*p ≤ 0.05).

## Results

### Fatty acid oxidation increased during the *in vitro* differentiation of osteoclasts

**FIGURE 1:**
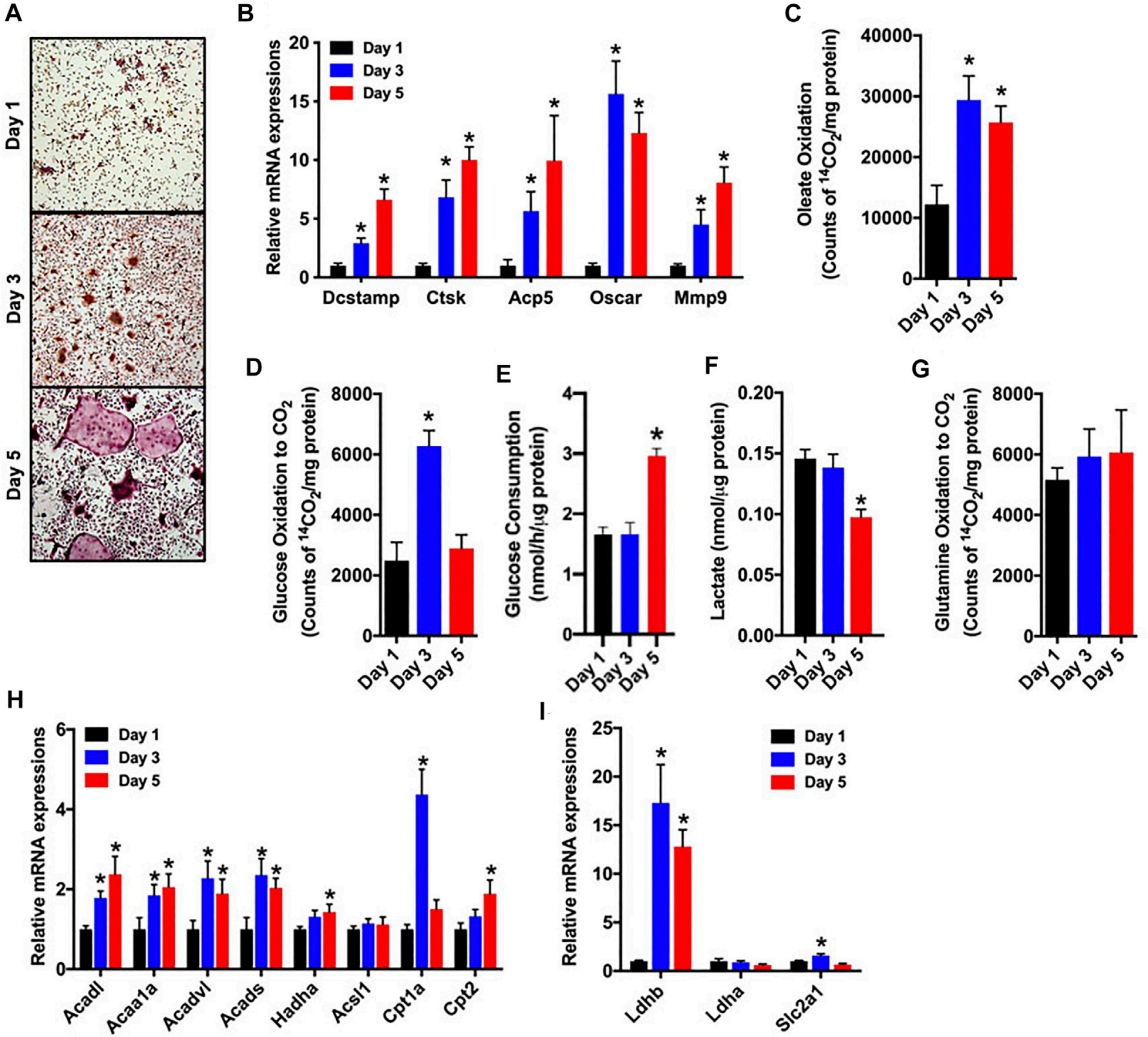
Fatty acid and glucose oxidation increase during osteoclast differentiation. Bone marrow cells were treated with M-CSF and RANKL to induce osteoclast differentiation *in vitro*. **(A,B)** TRAP staining (×10 original magnification) **(A)** and qPCR analysis of osteoclastic gene expression **(B)** were assessed on days 1, 3 and 5 of culture. Oxidation of [^14^C]-oleate to ^14^CO_2_ **(C)**, oxidation of [^14^C]-glucose to ^14^CO_2_ **(D)**, glucose consumption **(E)**, cellular lactate levels **(F)**, and [^14^C]-glutamine oxidation to ^14^CO_2_ **(G)** were assessed in differentiating osteoclasts. The mRNA levels of genes associated with fatty acid **(H)** and glucose metabolism **(I)** were assessed by qPCR. All data are represented by mean ± SEM. *p < 0.05 vs. Day 1. *n* = 5–7 for each time-point.

As a first step in examining metabolic activity during osteoclast differentiation, bone marrow cells were isolated from the femurs of C57BL/6 mice and treated with M-CSF and RANKL to promote osteoclastogenesis. Over the course of a 5-days experiment, the abundance of multinucleated, tartrate-resistant acid phosphatase-positive (TRAP) osteoclasts (Figure 1A) and the expression of gene markers of osteoclast maturation increased as expected (Figure 1B). In parallel, cultures stimulated for 1, 3 or 5 days were incubated with ^14^C-labelled substrates and the production of ^14^CO_2_ was used to index catabolic activity. Notably, the ability to oxidize oleate more than doubled between day 1 and day 3 (Figure 1C) and was accompanied by increases in the mRNA levels of multiple enzymes involved in mitochondrial long chain fatty acid β-oxidation (Figure 1H). Glucose oxidation (Figure 1D) and the expression of Glut1 (Slc2a1, Figure 1I) were increased on day 3, but then decreased with further differentiation. By contrast, glucose consumption from culture medium was only elevated on day 5 of differentiation (Figure 1E). Lactate levels (Figure 1F), an indicator of aerobic glycolysis, were not changed between days 1 and 3, but were reduced by more than 30% on day 5 when compared to day 1. Consistent with this finding the mRNA levels of Ldhb, which is thought to favor the conversion of lactate to pyruvate (Dawson et al., 1964), were increased on days 3 and 5. Glutamine oxidation did not change during the 5 day study (Figure 1G). Taken together these data highlight a dramatic increase in metabolic activity as osteoclasts differentiate *in vitro* and suggest that β-oxidation of fatty acids and to a lesser extent glucose oxidation are used to fuel osteoclast development. Differences in the timing of heighted glucose consumption and changes in glucose oxidation and lactate production, suggest that glucose may be utilized by osteoclasts in non-ATP generation pathways such as the pentose phosphate pathway or biosynthetic pathways.

### Inhibition of fatty acid β-oxidation in osteoclasts increases cancellous bone volume in female mice

**FIGURE 2:**
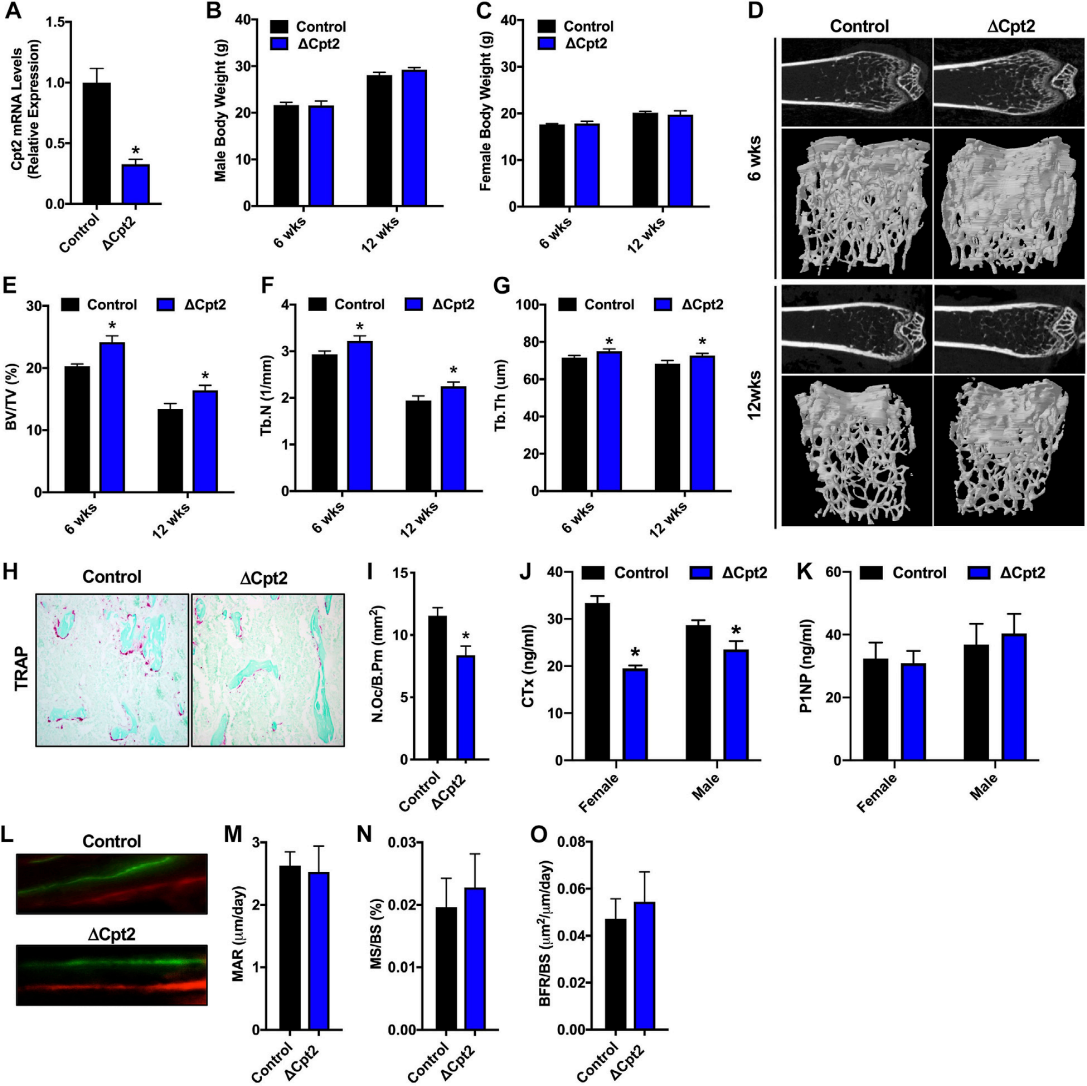
Female ΔCpt2 mice have increased bone mass with reduced numbers of osteoclasts. **(A)** qPCR analysis of Cpt2 mRNA levels in osteoclasts differentiated from bone marrow cells isolated from 6 week old control and ΔCpt2 mice. **(B,C)** Body weight was assessed in male **(B)** and female **(C)** control and ΔCpt2 mice at 6 and 12 weeks of age (*n* = 8–10 mice). **(D)** Representative computer renderings of bone structure in the distal femur of 6 and 12 weeks old female control and ΔCpt2 mice. **(E–G)** Quantification of bone volume per tissue volume (BV/TV, **(E)**, trabecular number (Tb.N, **(F)** and trabecular thickness (Tb.Th, **(G)** in the distal femur by microCT (*n* = 8–11 mice). **(H)** Representative staining for TRAP activity in 5 μm sections from the distal femur of 6 weeks old female mice (×10 original magnification). **(I)** Quantification of osteoclast numbers per bone perimeter (N.Oc/B.Pm) in the distal femur of 6 weeks old female mice (*n* = 6–7). **(J–K)** Serum analysis of CTx **(J)** and P1NP **(K)** in 6 weeks old male and female control and ΔCpt2 mice (*n* = 8–9 mice). **(L)** Representative dynamic histomorphometric images of female control and ΔCpt2 femurs labeled with calcein and alizarin red. Mineral apposition rate [MAR, **(M)**], mineralizing surface per bone surface [MS/BS, **(N)** and bone formation rate [BFR/BS, **(O)]** was assessed in the femurs of 6 weeks old female mice (*n* = 6–7). All data are represented by mean ± SEM. *p < 0.05.

Since fatty acid catabolism by the osteoclast has been poorly examined, we explored the requirement for mitochondrial long chain fatty acid β-oxidation in the osteoclast by examining bone structure in mice lacking *Cpt2* in myeloid cells (Cpt2^flox^; Lysozyme2-Cre, hereafter referred to as ΔCpt2) (Gonzalez-Hurtado et al., 2017). CPT1 is the rate-limiting enzyme in β-oxidation, but three isoforms are present in mammalian genomes that can exhibit functional compensation (Britton et al., 1995; Yamazaki et al., 1996; Price et al., 2002; Haynie et al., 2014). CPT2 is an obligate enzyme in long chain fatty acid catabolism and is encoded by a single gene. To ensure that Cpt2 expression is effectively ablated in the osteoclasts of ΔCpt2 mice, bone marrow cells isolated from control and ΔCpt2 mice were stimulated *in vitro* with RANKL and M-CSF for 5 days. Osteoclast cultures isolated from ΔCpt2 mice exhibited a 67% reduction in Cpt2 mRNA levels relative to control littermates (Figure 2A). The mutant mice exhibited normal body weight (Figures 2B,C) and previous studies documented that body composition, lipid homeostasis, and glucose tolerance were identical to controls (Gonzalez-Hurtado et al., 2017).

The effects of Cpt2 ablation in osteoclasts on bone structure exhibited a striking sexual dimorphism. At 6 and 12 weeks of age, trabecular bone volume in the distal femur of male control and ΔCpt2 mice were identical (Table 2). However, trabecular bone volume was consistently increased in female mutants relative to control littermates (Figures 2D,E) and was accompanied by corresponding increases in trabecular number (Figure 2F) and trabecular thickness (Figure 2G). The relative increase in bone volume was similar at both ages (+19.2% at 6 weeks of age vs. +22.5 at 12 weeks of age). Cortical bone structure at the femoral mid-diaphysis was not affected in Cpt2 mutants of either sex (Table 3).

**TABLE 2:** MicroCT and histological analysis of trabecular bone in the distal femur of male mice.

Bone parameter^a^	6 weeks		12 weeks	
	Control^b^	ΔCpt2	Control	ΔCpt2
Bone volume/tissue volume (%)	39.67 ± 2.57	38.51 ± 3.42	39.39 ± 0.81	36.27 ± 1.08
Trabecular number (1/mm)	4.46 ± 0.20	4.23 ± 0.20	4.16 ± 0.09	3.98 ± 0.12
Trabecular thickness (μm)	88.90 ± 4.58	89.60 ± 5.52	94.93 ± 1.81	91.27 ± 0.88
Trabecular spacing (μm)	155.51 ± 6.05	167.31 ± 9.65	169.14 ± 3.97	179.98 ± 5.56
Osteoclast number (N.Oc./B.Pm)	4.90 ± 0.84	7.18 ± 1.55	ND	ND

a Values are shown as Mean ± SEM.

b *n* = 9–13 for microCT, *n* = 5 for histology.

**TABLE 3:** MicroCT analysis of cortical bone structure in 12 Weeks old mice.

Bone parameter^a^	Female		Male	
	Control^a^	ΔCpt2	Control	ΔCpt2
Tissue area (mm^2^)	1.63 ± 0.02	1.57 ± 0.0.4	2.30 ± 0.05	2.32 ± 0.07
Cortical area/tissue area (%)	51.53 ± 0.57	52.62 ± 0.49	49.57 ± 0.04	49.43 ± 0.98
Cortical thickness (mm)	0.20 ± 0.01	0.20 ± 0.01	0.22 ± 0.01	0.22 ± 0.01

a Values are shown as Mean ± SEM.

a *n* = 9–13.

To uncover the cellular basis for the increase in trabecular bone volume in female ΔCpt2 mice, we preformed serum analyses of bone turnover markers, TRAP staining to assess osteoclast numbers and dynamic histomorphometric analyses to assess osteoblast function. At 6 weeks of age, the abundance of TRAP^+^ osteoclasts on trabecular bone surfaces was significantly reduced in female ΔCpt2 mice (Figures 2H,I) and those cells present in mutant mice were noticeably smaller than those in control mice. Likewise, serum levels of the C-telopeptide of type I collagen (CTx), a marker of bone resorption, was reduced by 41% in female mutants (Figure 2J). TRAP staining of femurs from 6 week old male mice revealed a trend towards increased osteoclast numbers per bone perimeter (Table 2), but a small decrease in serum CTx levels (−18%) was still observed in mutants, suggesting that these cells are functionally impaired. Osteoblast function was not affected by ablation of Cpt2 in osteoclast lineage cells as serum levels of N-terminal propeptide of type I collagen were similar in control and mutant mice from both sexes (Figure 2K). Further, the mineral apposition rate, the mineralizing surface per bone surface and the bone formation rate in the trabecular bone compartment was identical in female control and ΔCpt2 mice (Figures 2L–O). Therefore, mitochondrial long chain fatty acid β-oxidation is required for normal osteoclast development and postnatal bone homeostasis especially in growing female mice.

### Fatty acid β-oxidation is an important regulator of osteoclast differentiation *in vitro*

**FIGURE 3:**
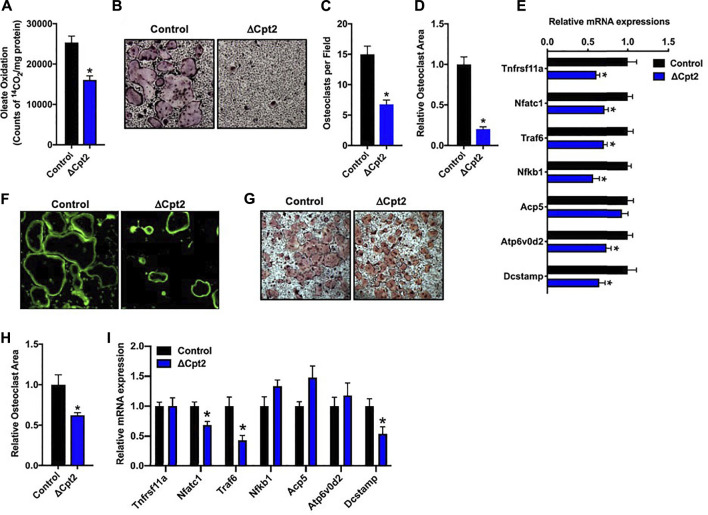
Mitochondrial fatty acid β-oxidation is required for osteoclast differentiation *in vitro*. Bone marrow cells isolated from female 6 weeks old control and ΔCpt2 mice were differentiated in the presence of M-CSF and RANKL for 5 days. **(A)** β-oxidation was indexed by the oxidation of [^14^C]-oleate to ^14^CO_2_. **(B)** Representative micrographs of TRAP-positive osteoclasts differentiated from control and ΔCpt2 bone marrow cells (×10 original magnification). Multinucleated (>3nuclei/cell) osteoclast per field **(C)**, relative osteoclast area **(D)** and relative mRNA levels of osteoclast differentiation associated genes **(E)** were assessed after 5 days of differentiation. As an indicator of osteoclast function, bone marrow cells were differentiated for ×5 days on Osteo-Assay plates to examine resorption pit formation [**(F)**, ×10 original magnification] or differentiated on tissue culture plastic and stained with FITC-phalloidin to examine actin ring formation [**(G)**, ×10 original magnification]. TRAP staining [**(H)**, ×4 original magnification] was performed and relative osteoclast area **(I)** was measured in osteoclast differentiated from bone marrow cells isolated from 6 weeks old male control and ΔCpt2 mice. All data are represented by mean ± SEM. *, *p* < 0.05. *n* = 4–10 for each condition.

To more closely examine the impact of inhibiting long chain fatty acid β-oxidation on osteoclast development, we differentiated osteoclasts from cultures of bone marrow cells isolated from female control and ΔCpt2 mice. Importantly, the inhibition of Cpt2 expression (Figure 2A) resulted in a 40% reduction in the ability to oxidation ^14^C-oleate to ^14^CO_2_ relative to control cultures (Figure 3A). The remaining capacity to oxidize oleic acid could be due to peroxisomal fatty acid oxidation, ω-oxidation or the presence of non-myeloid cells in the cultures.

Consistent with the osteoclast phenotype *in vivo*, the loss of Cpt2 function markedly impaired osteoclast formation *in vitro*. When compared to controls, the abundance of TRAP^+^ osteoclasts was substantially reduced in ΔCpt2 cultures and the multi-nucleated cells that were able to form in mutant cultures were significantly smaller (Figures 3B–D). The mRNA levels of key osteoclastic genes including Rank, Nfatc1, Traf6, Nfkb1, and Atp6vod2 (Figure 3E) were also reduced in ΔCpt2 cultures relative to controls of However, phallodin-staining (Figure 3F) revealed that the small number of osteoclasts evident in ΔCpt2 cultures were still able to polarize and form an actin ring. Thus, the loss of Cpt2 function appears to primarily result from a defect in osteoclast formation rather than a defect in polarization.

Osteoclast differentiation was also diminished in cultures of cells isolated from male ΔCpt2 mice (Figures 3G–I), but not to the extent that was observed in cultures of mutant cells isolated from females (Figure 3B). Here, bone marrow cells isolated from male mice formed an abundance of small osteoclasts, but failed to form the large osteoclasts evident in control cultures. These data are congruent with the trend towards an increase in the number of osteoclasts observed *in vivo* in the male mutant mice (Table 2) as well as the defect in the formation of large multinucleated cells evident in cell cultures isolated from female mutants.

### Loss of Cpt2 function cannot be rescued by glucose and stimulates the activation of AMPK

**FIGURE 4:**
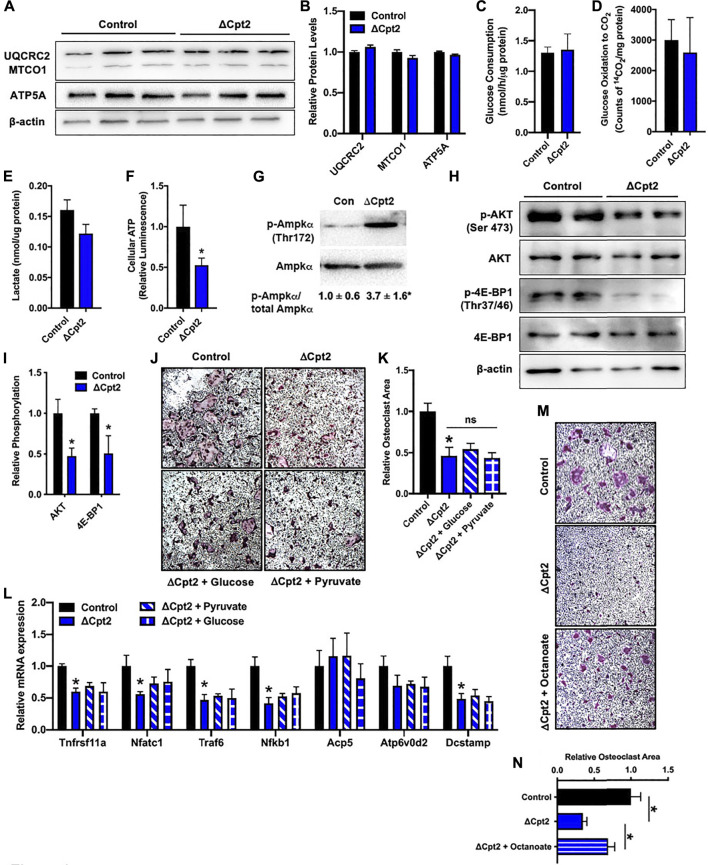
Impaired osteoclast differentiation in ΔCpt2 is associated with reduced ATP levels and cannot be rescued by exogenous glucose. Bone marrow cells from 6 weeks old, female control and ΔCpt2 mice were differentiated in the presence of M-CSF and RANKL for 5 days. **(A,B)** Representative immunoblot and quantification of mitochondrial proteins UQCRC2, MTCO1, and ATP5A in control and ΔCpt2 osteoclast cultures. Glucose consumption **(C)**, oxidation of [^14^C] glucose to ^14^CO_2_ **(D)** and cellular lactate levels **(E)** were assessed after 5 days of differentiation. **(F)** Relative cellular ATP levels were assessed in control and ΔCpt2 osteoclasts after 5 days of differentiation and normalized to protein concentration. **(G–I)** Immunoblot analysis and quantification **(I)** of AMPKα phosphorylation **(G)**, and the phosphorylation of mTOR targets AKT and 4E-BP1 **(H)**. Immunoblots were quantified using ImageJ with the levels of phospho-protein normalized to total protein levels. **(J)** Representative micrographs of TRAP + osteoclasts and **(K)** quantification of osteoclast area after differentiation in basal medium or medium supplemented with additional glucose (0.5 mg/ml) or pyruvate (1 mM). **(L)** qPCR analysis of osteoclastic gene expression in cultures grown in basal media or supplemented with glucose or pyruvate. **(M)** Representative micrographs of TRAP + osteoclasts and **(N)** quantification of osteoclast area after differentiation in basal medium or medium supplemented with octanoate (0.2 mM). All data are represented by mean ± SEM. *, *p* < 0.05. *n* = 4–8 for each condition.

Since manipulating the activity of one metabolic pathway often leads to compensatory changes in the activity of other pathways, we next questioned whether diminishing fatty acid β-oxidation altered the utilization of glucose by osteoclasts *in vitro*. We expected that mutant osteoclasts would maintain cellular ATP levels *via* an increase in glucose utilization as the expression profile of mitochondrial proteins, including UQCRC2, MTOC1, and ATP5A (Figures 4A,B), were similar in cultures of control and ΔCpt2 osteoclasts. However, neither glucose consumption (Figure 4C), oxidation of ^14^C-labelled glucose to ^14^CO_2_ (Figure 4D) or lactate levels (Figure 4E) were increased in ΔCpt2 osteoclast cultures when compared to controls. As a result, ΔCpt2 cultures exhibited a significant reduction in cellular ATP levels (Figure 4F). Additionally, the mutant osteoclast cultures exhibitied a significant increase in the phosphorylation of AMPKα (Figure 4G) and reduce phosphorylation the mTOR targets AKT and 4E-BP1 (Figures 4H,I), which is indicative of an energy stress response (Inoki et al., 2003).

We also attempted to rescue the phenotype of ΔCpt2 cells by increasing the concentration of pyruvate or glucose in the culture medium by 50% differentiation. However, the addition of these energy sources had little effect on either osteoclastic TRAP staining or gene expression (Figures 4J–L). By contrast, the addition of octanoate, a medium chain fatty acid that can be oxidized independent of CPT1 and CPT2 (Guo et al., 2006; Pereyra et al., 2021), partially rescued the effect of CPT2-deficiency on TRAP staining (Figures 4M,N). Taken together, these data highlight a critical role for fatty acid oxidation in the maintenance of cellular energetics during osteoclast differentiation and indicate that glucose metabolism is unable to compensate for the loss of fatty acids as an energy source.

## Discussion

The metabolic requirements of the osteoclast and the preferred fuel source as myeloid lineage cells commit to the osteoclast lineage and fuse to form multinucleated osteoclasts responsible for bone resorption are still poorly understood. Exploration of these requirements will deepen our understanding of the mechanisms that control osteoclast development and function and may ultimately lead to additional strategies to influence osteoclast activity and combat bone loss. In this work, we examined the necessity for mitochondrial long chain fatty acid β-oxidation during osteoclast development by disrupting the expression of *Cpt2* in the myeloid-macrophage lineage (Clausen et al., 1999; Gonzalez-Hurtado et al., 2017). We find that female mutants exhibit an increase in trabecular bone volume with reduced numbers of TRAP + osteoclasts *in vivo* and that the differentiation of Cpt2-deficient osteoclasts is severely impaired *in vitro*.

Mitochondrial respiration appears to play a key role in meeting the energetic needs of the osteoclast. Histological studies report that the abundance of mitochondria increases as progenitor cells fuse (Chuan, 1931), while more recent molecular studies have found that RANKL induces mitochondrial biogenesis and that the transition from bone marrow macrophage to TRAP + osteoclast is accompanied by an increase in the expression of genes encoding components of the mitochondrial respiratory complexes (Ishii et al., 2009; Miyazaki et al., 2012; Zeng et al., 2015). Importantly, ablating the expression of *Tfam*, a transcription factor necessary for mitochondrial gene expression, in osteoclasts dramatically reduced osteoclast numbers *in vivo* and was associated with reduce ATP levels and increased apoptosis after cytokine withdraw *in vitro* (Miyazaki et al., 2012). Likewise, global or hematopoietic deficiency for NDUFS4, critical for the assembly of mitochondrial respiratory complex I, resulted in an osteopetrotic phenotype and impaired osteoclast differentiation (Jin et al., 2014).

Consistent with an increase in oxidative metabolism during osteoclast differentiation, we observed a dramatic increase in long chain fatty acid oxidation between day 1 and day 3 of osteoclast differentiation and a high capacity for fatty acid oxidation through day 5 of *in vitro* culture. These data as well as the increase in the mRNA levels of enzymatic mediators of fatty acid oxidation accord with the increased abundance of CPT2, ACADM, ACADS, ACADVL, and HADHA protein reported in a proteomic analysis of RAW264.7 cell differentiation to osteoclast (Xiong et al., 2016). Determination of the transporters by which osteoclasts and their precursors take up fatty acids will require additional study, but modulation of CD36 activity has been show to inhibit osteoclast activity *in vitro*, though this may at least partially be due to altered thrombospondin signaling (Dawodu et al., 2018; Koduru et al., 2018).

Bone marrow cells isolated from female ΔCpt2 mice formed fewer and smaller osteoclasts after RANKL stimulation when compared to cells isolated from control littermates. Due to the severity of this phenotype and the fact that osteoclasts in ΔCpt2 mice appeared smaller in TRAP stained sections, fatty acid oxidation appears to fuel cellular fusion events that are necessary to form large osteoclasts (Oursler, 2010). Indeed, phallodin staining revealed that the small number of multinucleated osteoclasts formed in ΔCpt2 cultures were able to polarize and form an actin ring. This finding is consistent with studies performed in macrophages, wherein the inhibition of fatty acid oxidation *via* Cpt2 gene ablation did not inhibit polarization after cytokine stimulation (Nomura et al., 2016; Gonzalez-Hurtado et al., 2017). Surprisingly, supplemental glucose or pyruvate was not able to rescue the effect of Cpt2 loss of function on osteoclast differentiation. We observed a transient increase in glucose oxidation during the differentiation of wildtype cells to osteoclasts and a number of studies have documented the importance of this energy source during osteoclast differentiation (Lu et al., 2001; Ahn et al., 2016; Li et al., 2020). Since inhibiting glucose uptake *via* the Glut1 transporter impairs osteoclast differentiation (Li et al., 2020) and we previously observed a compensatory increase in glucose utilization following *Cpt2* ablation in osteoblasts (Kim et al., 2017), we expected to observe an increase in glucose utilization in ΔCpt2 osteoclasts or at least a reduction in the severity of the phenotype with an increase in available glucose. Instead, cultures of mutant osteoclasts exhibited a reduction in cellular ATP levels and an increase in the phosphorylation/activation of AMPKα, an inhibitor of osteoclast differentiation (Lee et al., 2010; Tong et al., 2020). In turn, ΔCpt2 cultures displayed a reduction in the phosphorylation status of mTOR targets that are expected to contribute to the regulation of osteoclast fusion and cytoplasmic growth (Cejka et al., 2010; Tiedemann et al., 2017; Tong et al., 2018). Taken together these data suggest that fatty acid oxidation is essential for normal osteoclast formation *in vitro*.

The sexually dimorphic skeletal phenotype observed in ΔCpt2 mice, with female but not male mutants exhibiting an increase in trabecular bone and reduced osteoclast numbers, was surprising, but not entirely unexpected. Mun and colleagues (Mun et al., 2021) queried differences in the transcriptional profiles of osteoclasts isolated from female and male mice and found differential expression of genes involved in the NF-κB-NFATc1 axis that drives osteoclast differentiation (though enrichment in metabolic genes was not found). Additionally in our previous work, Cpt2-deficiency in osteoblasts also produced a more severe defect in trabecular bone architecture in female mice (Kim et al., 2017). We reasoned that sexual dimorphism in the osteoblast knockout model may be due to an inability to modify fuel selection in female mice because estrogen favors fatty acid metabolism (Hatta et al., 1988; Kendrick and Ellis, 1991; Campbell and Febbraio, 2001). Though not directly examined here, the same effect could be responsible for the phenotype in osteoclast-specific mutants. The reduced number of osteoclasts in female ΔCpt2 mice may result from the combined effect of impaired metabolism and estrogen’s ability to shorten the lifespan of osteoclasts by inducing apoptosis (Nakamura et al., 2007; Martin-Millan et al., 2010). Consistent with this idea, Kim and colleagues (Kim et al., 2020) reported that estrogen reduces oxidative metabolism in osteoclast precursors through the down-regulation of genes involved in the formation of mitochondrial complex I. Male ΔCpt2 mutants exhibited a trend towards increased numbers of osteoclasts *in vivo* (a phenotype that was also apparent *in vitro* with the accumulation of small osteoclasts), which raises the possibility that fuel selection is able to be adjusted or another compensatory mechanism is engaged to maintain a balance in bone remodeling. The linkage between sex hormones and the intermediary metabolism of the osteoclast is deserving of further study.

In summary, our data suggest a critical role for fatty acid metabolism during osteoclast differentiation and in normal bone remodeling, especially in female mice. It will be of interest in future studies to determine if this requirement in growing mice changes with age or under conditions of hormone-deficiency.

## Data Availability

The raw data supporting the conclusion of this article will be made available by the authors, without undue reservation.
